# Altered Volcanic Tuffs from Los Frailes Caldera. A Study of Their Pozzolanic Properties

**DOI:** 10.3390/molecules26175348

**Published:** 2021-09-02

**Authors:** Jorge Luis Costafreda, Domingo Alfonso Martín, Leticia Presa, José Luis Parra

**Affiliations:** 1 Escuela Técnica Superior de Ingenieros de Minas y Energía, Universidad Politécnica de Madrid, Calle Ríos Rosas 21, 28003 Madrid, Spain; domingoalfonso.martin@upm.es (D.A.M.); leticia.presa.madrigal@alumnos.upm.es (L.P.); joseluis.parra@upm.es (J.L.P.); 2 Laboratorio Oficial para Ensayos de Materiales de Construcción—LOEMCO, Calle Eric Kandel 1, 28906 Madrid, Spain

**Keywords:** altered volcanic tuff, pozzolanicity, mortar, cement, mechanical strength

## Abstract

This work presents the results of the study of the physical, chemical, mineralogical and pozzolanic properties of the altered volcanic tuffs (AVT) that lie in the Los Frailes caldera, south of the Iberian Peninsula, and demonstrates their qualities as pozzolans for the manufacturing of mortars and pozzolanic cements of high mechanical strength. The main objective of this research is to show to what extent the AVTs can replace portland cement (PC) in mortars, with standardised proportions of 75:25% and 70:30% (PC-AVT). To achieve these objectives, three AVT samples were studied by a petrographic analysis of thin section (PATS), DRX, FRX and MEB. The pozzolanic properties were determined by three methods: electrical conductivity (ECT), chemical pozzolanicity tests (CPT) at 8 and 15 days and mechanical strength tests (MS) of the specimens at 2, 7, 28 and 90 days. Studies of a PATS, DRX, FRX and MEB showed that the AVT samples’ constitutions are complex where smectite (montmorillonite), mordenite, quartz, halloysite, illite, kaolinite, volcanic glass and lithic fragments coexist. The results of the ECT and CPT tests confirmed the pozzolanic properties of the samples analysed and proved an increase in mechanical strength from 2 to 90 days of testing.

## 1. Introduction

Many natural materials are currently used as pozzolanic agents aimed at improving the quality of cements, mortars and concretes [1,2,3,4], with low production costs and with good CO_2_ emission mitigating properties, [5,6,7], the reduction of hydration heat [8] and the increase of resistance to attack by external agents on construction structures [9]. These natural materials are widely distributed in the Earth’s crust, and their exploitation is carried out according to sustainable geological and mining parameters [10]. In recent decades, the knowledge of natural pozzolans has increased markedly [11], as well as the prospecting procedures that control selective exploitation [12]. Other problems have been seen in the production process of companies that focus on technological properties [13] rather than on the intrinsic characteristics of these materials [14,15,16,17]. The use of tuff as a pozzolan has increased in recent decades in many parts of the world [18,19,20,21,22], with well-known results. Volcanic tuffs can match and outperform other minerals and industrial rocks, such as zeolites, bentonites, perlites, pumicites, trachytes, rhyolites and kaolinites [23,24,25,26,27,28], with regards to their cementing properties.

Mustapha et al. [29] performed studies to improve the performance of local materials, such as tuffs and dune sands, for use in road construction. The results of their investigations showed that tuffs are suitable for base layers and foundations. Tuffs have been used as aggregates replacing 25% of the traditional aggregate in brick manufacture, according to Kamel et al. [30]. Some researchers [31] have designed concrete mixes with calcareous tuffs, which partially replace fine aggregates (sand) by 25%, achieving nearly 33% mechanical strength. Ababneh and Matalkah [32] replaced portland cement with various types of volcanic tuffs at 10–40% by weight; they established that the more siliceous tuffs contribute more mechanical strength at early ages, while the more calcic tuffs provide more mechanical strength in the long term. In the formulation of fibre concretes, tuffs have played an important role, as demonstrated by Ruslan et al. [33]. These mixtures are suitable for the construction and restoration of buildings. It is widely known that zeolitic tuffs are commonly used in pozzolanic cements and mortars and are of vital importance to local industries in many countries [34]. Volcanic tuff horizons in the Alps have played an important role in the tephrostratigraphic correlation of the Middle Miocene in Europe, as described by Rocholl et al. [35]. Some researchers, such as Balegh et al. [36], have succeeded in improving the properties of tuffs by mixing them with pulverised ceramic waste in different proportions. According to their findings, the higher the addition of ceramic waste, the higher the mechanical strength and the better the geotechnical properties. Sarireh [37] developed high-performance concretes with 20% tuff replacing gravel and sand, obtaining high mechanical strengths. In the work of Capaccioni et al. [38], volcanic tuffs are analysed as a means of predicting lung cancer risks based on some inherent properties of these rocks, such as: radionuclide content, emanation power and air exchange rate, among others. The study of the grain/matrix ratio of some tuffs, as proposed by Korkanç and Solak [39], is suitable for the estimation of their engineering and geomechanical properties. Volcanic tuffs have been used in the construction of historic buildings and monuments that are preserved despite alterations caused by weathering processes [40]. Furthermore, Heap et al. [41] determined that the weathering of these monuments is caused by the presence of zeolite in the tuffs. Some recent studies [42] described the use of clinoptilolitic tuff as a foaming additive in asphalt technology, capable of replacing ordinary filler with positive results.

The work carried out in this research has provided novel and relevant results that increase the interest and perspective of the altered volcanic tuffs found in the Los Frailes caldera, which could be used as local quality products for the manufacture of pozzolanic cements, concretes and mortars.

## 2. Results and Discussion

### 2.1. Results of Petrographic Analysis by Thin Sections (PATS)

**Figure 1:**
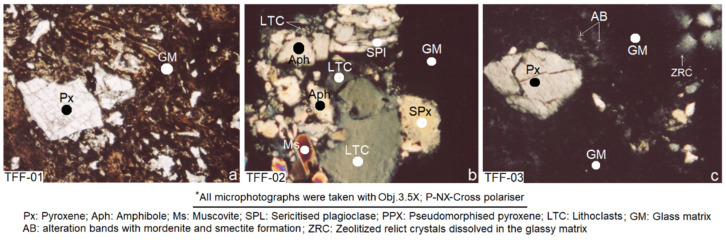
(**a**–**c**) Petrographic thin-section microphotographs taken during the study of the researched samples.

The petrographic study established that the researched samples have a very complex mineralogical composition, made up mostly of devitrified volcanic glass, clasts and epiclastic lithics, broken fragments of pyroxene, amphibole, plagioclase and quartz (Figure 1a–c). The matrix is mostly vitreous, devitrified and glomeroporphyritic black to yellowish-brown, as seen with crossed polarisers. Note how, in Figure 1a, pyroxene phenocrystals have undergone a process of zeolitization and acquired lighter colours. The matrix retains the features of stratification at the time of the deposition of ash, crystal clasts and lithic epiclasts in the form of small planes or sinuous bands. The process of zeolitization of the phenocrystals was possibly produced by the penetration of fluids from the hydrothermal alteration of the volcanic glass, which circulated through the planes of cruises of pyroxenes and amphiboles; this deduction seems to make sense when compared to the conclusions of References [43,44,45].

Figure 1b shows a vitreous matrix in which angular, subangular and subrounded self-supported lithoclasts of green to greenish-grey of varying sizes are observed. Next to these fragments are phenocrystals of plagioclase sericitised and smectitised, in addition to muscovite and quartz. Figure 1c has a broken and isolated pyroxene phenocrystal, pseudo-morphically replaced by zeolite and embedded in a predominantly vitreous matrix. Halos of light colour can be seen in various parts of the matrix, which indicates their transformation into smectite and mordenite. Some protominerals in the upper right end of the microphotograph have reacted almost completely with the matrix.

As can be observed, the samples studied have a complex texture type of glass–crystal–lithic–clastic and are banded. Their formation is related to the pyroclastic processes that produced very fine materials of the cinerite type, as shown in Figure 1a–c. Observations of this type appear in the works of Costafreda J.L. [45]. A majority of the vitreous matrix and the presence of crystal–clasts (pyroxene and plagioclase), lithoclasts and secondary minerals (mordenite and montmorillonite) with a high specific surface area and a very small particle size are very important factors for this research, since they contribute to the pozzolanic reactivity of the samples analysed.

### 2.2. X-ray Diffraction (XRD) Analysis Results

**Figure 2:**
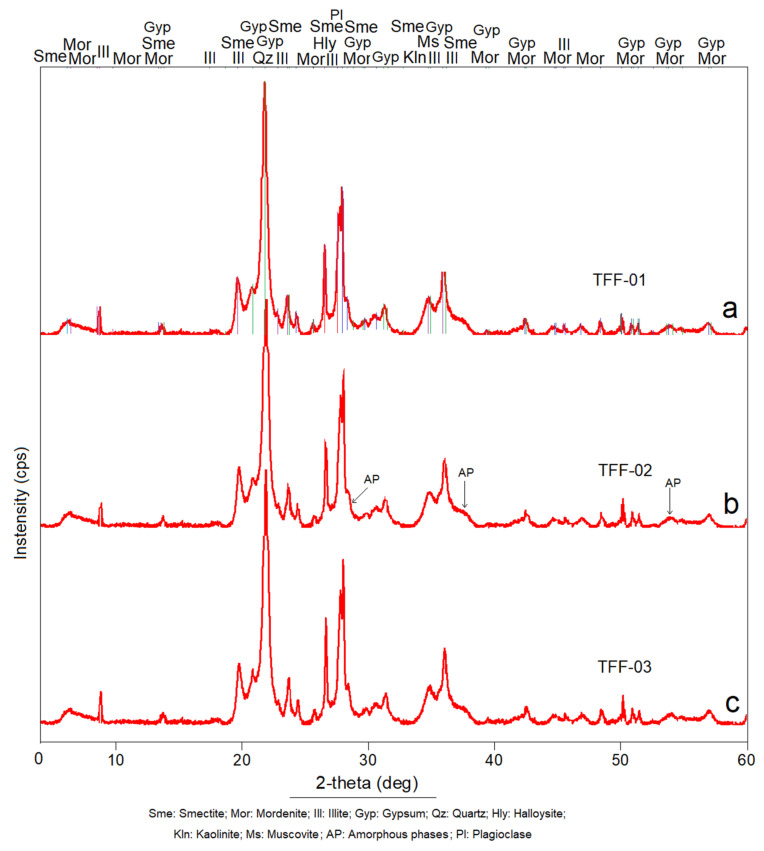
X-ray diffraction patterns of the analysed samples. (**a**) sample TFF-01, (**b**) sample TFF-02 and (**c**) sample TFF-03.

As seen in the previous section, the studies using XRD also confirmed the complex constitution of the samples seen in Figure 2. The appearance of X-ray diffraction patterns indicates the presence of well-defined crystalline phases in the form of intense and acute peaks originated by minerals with well-organised internal structures consisting of mordenite and plagioclase, which were mentioned in the discussion in the previous subsection and in Figure 1a–c.

The X-ray diffraction patterns also showed the amorphous phases (AP) of, mainly, altered volcanic glass, whereby the crystalline disorganisation does not produce strong peaks or intense reflections; instead, it forms a warped band in the background of the diffractogram, as seen in Figure 2b. Specifically, the mineralogical phases detected were the following: smectite (montmorillonite), mordenite, plagioclase, illite, gypsum, quartz and kaolinite, in addition to amorphous materials. In the Special Paper of the Geological Society of America [46] somewhat similar mineralogical phases have been described for tuffs of dacitic and rhyolitic composition, with which this work coincides quite well. The joint existence of these phases confirms the pyroclastic and volcano-sedimentary genesis of the samples, which agrees with the deductions made by the authors in this work and with the conclusions established by Costafreda [45], Soriano et al. [47] and Oyarzun et al. [48].

### 2.3. Results of the X-ray Fluorescence (XRF) Study

The results of the study of the chemical compositions of the samples are seen in Table 1. The anomalous values of SiO_2_ and Al_2_O_3_ in the three samples are highlighted; this is a fundamental characteristic of pozzolans; this assertion coincides with the conclusions researched by many authors, such as Christiansen and Dyamond [49]. Another aspect to highlight is the somewhat higher contents of alkaline compounds (Na_2_O and K_2_O) with regards to alkaline–earth (CaO and MgO). Specifically, small differences are observed between each sample, although the TFF-01 sample stands out for its SiO_2_ and Al_2_O_3_ contents, followed by TFF-03. This trend is manifested in the values of Na_2_O, K_2_O and Fe_2_O_3_, as well as in the loss on ignition (LOI). These traits are comparatively consistent with those described by Mark et al. [50]. An even closer similarity can be seen in the composition of the TFF-01 and TFF-03 samples, which may be caused by the influence of the zeolitization process, as confirmed by Costafreda [45] and Arribas [51]. However, the TFF-02 sample stands out for its slightly higher values in alkaline–earth compounds, such as CaO, MgO and Fe_2_O_3_, which indicate the presence of more clay minerals, such as montmorillonite, illite and kaolinite.

As stated above, the altered volcanic tuffs are host rocks of zeolitic mineralisation within the Los Frailes caldera, so their proximity favours a diffusive interaction between both geological formations. Thus, the TFF-01 and TFF-03 samples, closer to the points of highest zeolite concentration, have more silica, alumina, sodium oxide and potassium oxide and a greater loss on ignition (LOI) than the TFF-2, located in a more distal position and more affected by the smectisation process; therefore, in its chemical composition, magnesium oxide and iron contents stand out [52].

### 2.4. Scanning Electron Microscopy (SEM) Results

**Figure 3:**
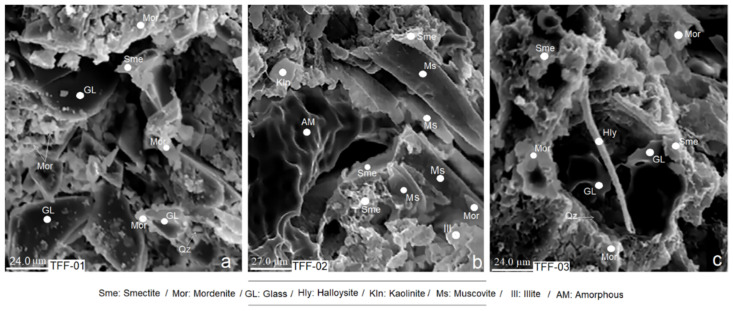
(**a**–**c**) Microphotographs of the analysed samples obtained by scanning electron microscopy.

The microphotographs shown in Figure 3a–c show a coalescence of crystal fragments, amorphous materials and minerals of secondary formations. Figure 3a shows crystals of mordenite, smectite and quartz, with a marked presence of volcanic glass. In Figure 3b, in addition to smectite and mordenite, kaolinite, illite, muscovite and amorphous materials are seen. In Figure 3c, there is halloysite, smectite, mordenite, quartz and volcanic glass.

Note that, in both Figure 3a and 3c, the presence of mordenite is greater than in Figure 3b; in the latter, the predominance of smectite is more evident. The explanation given is confirmed by what is discussed in Section 2.1 and Section 2.2. In all cases, secondary minerals (smectite, mordenite, illite and halloysite, among others) can be seen to grow from the volcanic glass [53]. An obvious example of this can be seen in Figure 3c, where a cylindrical halloysite crystal has grown freely from the altered volcanic glass.

### 2.5. Electric Conductivity Test (ECT) Results 

**Figure 4:**
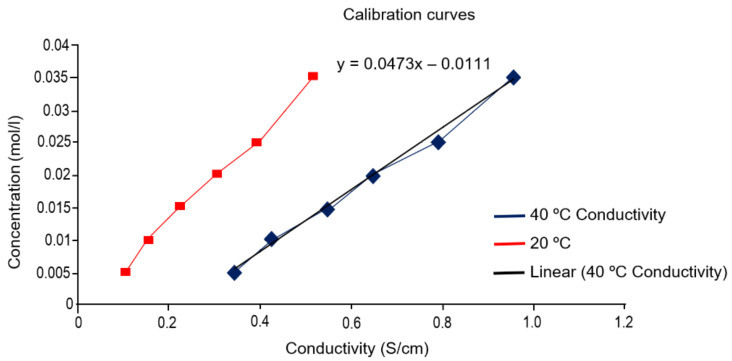
Curves conductivity patterns at different temperatures.

The graph in Figure 4 shows the relationship between conductivity and concentration by which the Ca^2+^ concentration and calibration curve were determined. The equation that characterises this correlation at 40 °C is as follows (1):

(1) y=0.0473x−0.0111

The correlation coefficient is (2):

(2) $$R^{2}=0.9949$$

which establishes the degree of linear dependence between the conductivity and concentration.

**Figure 5:**
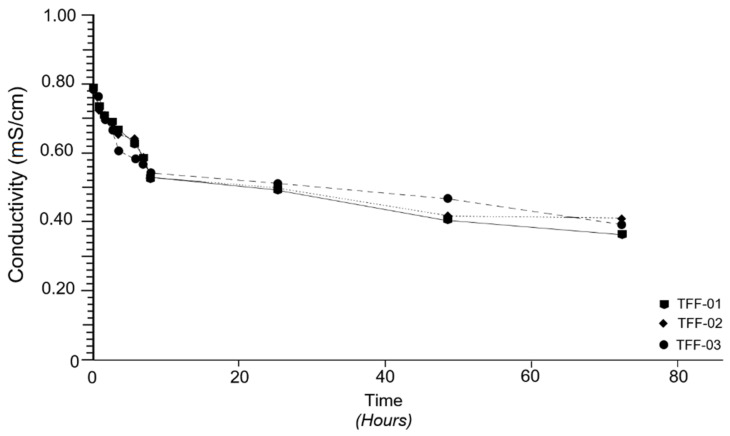
Evolution of the electrical conductivity at different time periods.

The concentration of Ca(OH)_2_ in different time periods was obtained by measuring the conductivity and by the evaluation of Equation (1). Figure 5 shows the pozzolanic reactivity of each of the samples analysed, according to the variations in electrical conductivity. A relative similarity in the behaviour of the samples is observed in the graph between 0 and 24 h with practically insignificant variations. In general, the values of electrical conductivity (EC) drop sharply from 0.78 to 0.52 mS/cm in the first 7 h, which is evidenced by the strong gradient seen in the respective curves in this time interval.

Between 7 and 24 h, a decrease in conductivity from 0.52 to 0.49 mS/cm is still evident, and between 24 and 48 h, this decrease is even more evident (0.49 to 0.40 mS/cm). The lowest values of electrical conductivity (EC) (0.36 mS/cm) were obtained after 72 h of testing.

Specifically, the pozzolanic reactivity of the TFF-01 sample is highlighted, which is able to transport more electrical charges and concentrate more Ca(OH)_2_ in the solution as the concentration decreases [54]. This is followed by the TFF-02 sample, which maintains its pozzolanic reactivity until 48 h, where its saturation is manifested in the form of a horizontal trajectory that is maintained up to 72 h; this is interpreted as a loss of pozzolanic reactivity due to saturation in Ca(OH)_2_ and the inability to carry more electrical charge. The TFF-03 sample, which was initially the least pozzolanic, appears to recover after 48 h, surpassing the TFF-02 sample.

**Figure 6:**
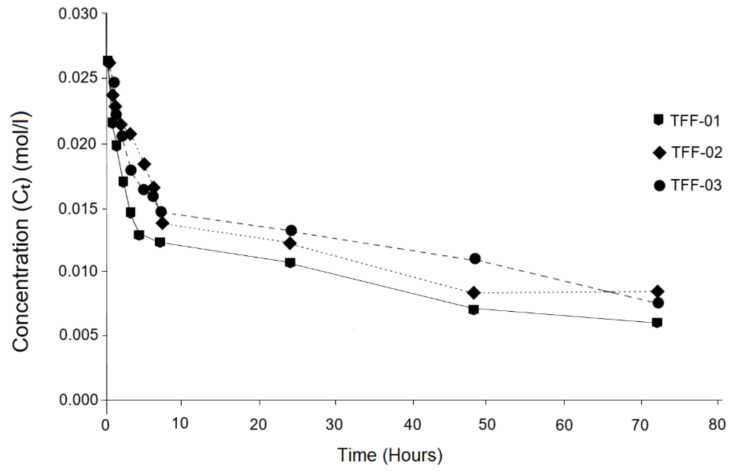
Evolution of the CaO concentration process in different time periods.

In the graph in Figure 6, there is a trend that is quite similar to that shown in Figure 5, which is interpreted as a directly proportional relationship between the electrical conductivity (EC) and the concentration of Ca(OH)_2_. Note that, in both graphs (Figure 5 and Figure 6), the main behavioural variations occur at the same time intervals, which has allowed to establish that variations in the concentration of Ca(OH)_2_ in the solution radically influence the behaviour of electrical conductivity (EC); that is to say, the decrease in concentration leads to a decrease in conductivity. It is evident that these variations are conditioned by the mineralogical, petrological and chemical constitutions of the samples analysed, as has already been discussed in the previous subsections. This means that the samples investigated behave like suitable natural pozzolans [54].

As seen in Figure 5, Figure 6 also shows the strong pozzolanic character of the TFF-01 sample in relation to the TFF-2 and TFF-03 samples; thus, it follows that, for a given time, some samples fix more Ca(OH)_2_ than others, indicating the degree of pozzolanic reactivity. Finally, the inclination that takes place in the curves of the TFF-01 and TFF-03 samples towards the *X*-axis is highlighted; this factor predicts the continuity of the pozzolanic reaction beyond 72 h.

### 2.6. Results of the Chemical Pozzolanicity Test (CPT)

**Figure 7:**
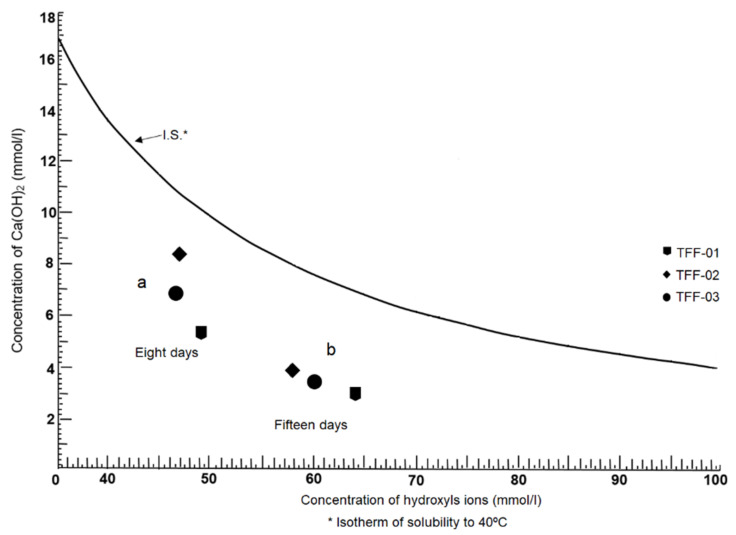
Variation of the pozzolanic behaviour of the samples analysed at 8 and 15 days. (**a**) samples analysed at 8 days; (**b**) samples analysed at 15 days.

The results of the chemical pozzolanicity test (CPT) are shown in Figure 7. According to this graph, it established that the three samples analysed have very marked pozzolanic properties, which manifest at both 8 and 15 days, as is seen in their positions under the solubility curve at 40 °C.

The locations of the samples in the graph after 8 days of testing (Figure 7a) show comparatively dispersed characters despite their evident reactivity in which the advantageous positions of the samples TFF-01 and TFF-03 are in relation to sample TFF-02. This trend can be extrapolated to what has already been discussed in other subsections, where the chemical composition and mineralogical constitution of the samples have a direct impact on their pozzolanic properties [55,56].

After 15 days of testing, the behaviour goes from dispersed to linear (Figure 7b). In this period, the samples adopt a linear and parallel ordering in the space between the solubility isotherm and the *X*-axis; this means that the process of pozzolanic reactivity becomes more regular as the reaction time elapses. Therefore, this research established that, despite the complex mineralogical, petrological and chemical constitutions of the samples, the pozzolanic properties seem to increase instead of decrease. The basis of this statement is evidenced by the presence of volcanic glass where its degree of devitrification and thermodynamic instability make it particularly reactive [15]; however, not only does glass provide these properties to the samples studied but, also, to mordenite, smectite (montmorillonite) and halloysite, which have inherent properties, such as the cation exchange capacity (CEC), sorption and extensive surface area reinforce pozzolanic reactivity. Another factor that influences the pozzolanic behaviour of the samples studied is the degree of fineness of the volcanic particles present where the active surface has been greatly increased. It is noteworthy that the deeper the location of the samples below the solubility isotherm, the greater their degree of pozzolanicity; that is to say, the greater the capacity of the sample to react with Ca(OH)_2_, which saturates the solution [57]. It can also be stated as the ability of the samples to fix more free lime in the reaction system depicted in the graph in Figure 7.

Both the electrical conductivity test (ECT) and the chemical pozzolanicity test (CPT) are efficient and adequate to establish the pozzolanic quality of the analysed samples, as discussed above in Section 2.4 and Section 2.5, respectively.

Many authors have described these processes in various types of natural pozzolans, such as mordenite and heulandite-clinoptilolite [58,59].

### 2.7. Results of the Mechanical Strength Test (MST)

**Figure 8:**
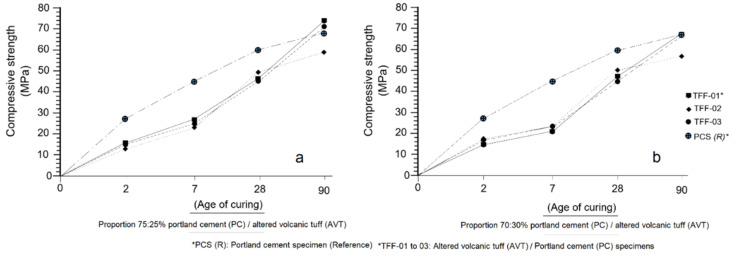
Mechanical compressive strength obtained at different curing ages. (**a**) 75:25% mixture ratio (PC:AVT); (**b**) 70:30% ratio (PC:AVT).

Figure 8a,b shows the results obtained by the means of the mechanical compressive strength test [60] carried out on the PC-AVT and PCS-R specimens. The graph in Figure 8a refers to the 75:25% mixture ratio (PC:AVT), while Figure 8b reports data from the 70:30% ratio (PC:AVT), respectively. As explained in Section 3.2, the tests were performed at different ages (2, 7, 28 and 90 days).

A detailed analysis of Figure 8a verifies the high hydraulic reactivity of portland cement (PCS-R) in relation to the specimens of AVT-PC (TFF-01–03), which indicates a high initial compressive strength between 2 (28 MPa) and 7 days (56 MPa) of curing. This situation prevails at greater ages (28 days 60 MPa), when the normal strengths of the specimens have been reached [61]; however, at 90 days of curing, the AVT-PC (TFF-01 and TFF-03) specimens are able to overcome the compressive strength (TFF-01 = 74.5 MPa and TFF-03 = 72.5 MPa, respectively) of the portland cement specimens (PCS-R = 68 MPa). The analysis of the behaviour of the TFF-01–03 specimens seems to indicate that the replacement of 25% of the PC in the mortar mixture does not appear to have a negative influence on the gain of mechanical strength throughout the test period, although this increase in strength is slow and delayed in relation to the PCS-R; this is one of the fundamental properties that pozzolanic materials have and has been widely reflected in the works of many authors [45,62,63,64]. The TFF-01 specimen curve shows more consistent behaviour than the TFF-02 and TFF-03 specimens.

The analysis of Figure 8b shows that the PCS-R behaves similarly to Figure 8a; however, the mechanical strength curves of the TFF-01–03 specimens show a slightly different configuration in the curing period of 2 to 7 days, which indicates that a 30% increase in AVT in the mixture slows the gain of mechanical strength at early curing ages even more. However, between 7 and 28 days, the curves recover quickly and acquire a higher gradient, which is interpreted as a reactivation of the hydraulic reaction system and, consequently, an increase in compressive strength. This behaviour has already been described by many authors in their research with different types of pozzolans, both natural and artificial [65,66]. The behaviour of the TFF-01 and TFF-03 specimens is highlighted, which equals the compressive strength of the PCS-R specimen at 90 days, despite replacing portland cement (PC) by 30%. According to the mechanical behaviour of the specimens there is almost no differences in the compressive strength between the proportions 75:25% and 70:30% (PC-AVT).

Finally, the porous and interconnected channel structure of the mordenite present in the AVTs analysed in this work should be taken into account for use in the manufacturing of pervious concrete [67], as it can provide permeability and strength. As discussed in Section 2.6, the degree of fineness of the sample also radically influences the hydraulic reaction process and the gain of mechanical strength, which is in line with Li et al. [68].

## 3. Materials and Methods

### 3.1. Materials

**Figure 9:**
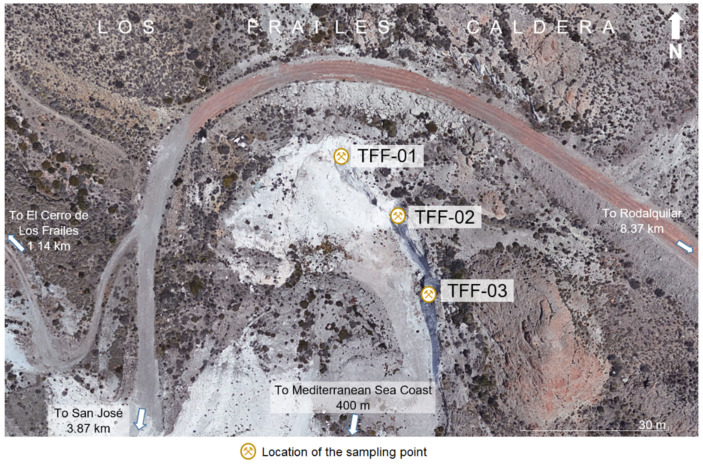
Location of the sampling points [69].

In this research, three samples of altered volcanic tuff (AVT) of dacitic composition (TFF-01, TFF-02 and TFF-03) were analysed, which were taken inside the Los Frailes caldera (Cabo de Gata, Almería, Spain). The investigated AVTs are the host rocks of the zeolitic mineralisation that lies inside the caldera. The location of the sampling points is given in Figure 9.

**Figure 10:**
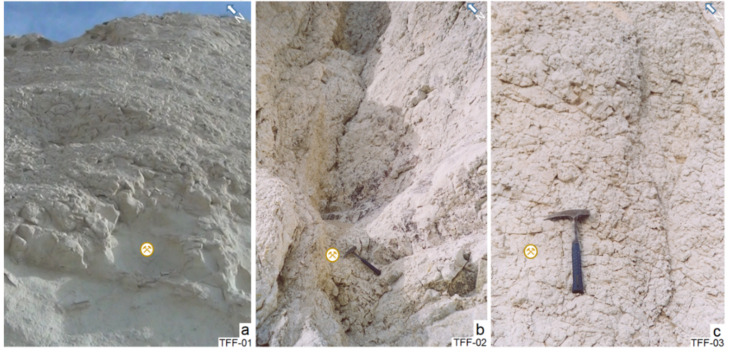
(**a**–**c**) View of the altered volcanic tuff outcrops (AVT) at the sampling points.

On the outcrops, AVTs vary in colours, from white, light grey, green and light brown to dark brown, and are occasionally covered by oxides of iron and manganese. They contain a breccian structure banded and stratified. These rocks are generally not very dense; they are light, powdery to the touch, fragmented and crossed by many cracks and diaclases. They are affected by spheroidal weathering (Figure 10a–c). In Figure 10a, the AVTs are comparatively thinner, cineritics and friable than those shown in Figure 10b,c, which have a thicker grain size. They are often altered into zeolites, bentonites or both.

### 3.2. Methods

#### 3.2.1. Petrographic Analysis of Thin Sections (PATS)

The thin-section petrographic study was carried out with a Leica DM600M Scope microscope equipped with a DTA-13 system of monochromator filters of visible and infrared light for 13 wavelengths, from 400 nm to 1000 nm, at intervals of 50 nm. The equipment has the Cameva System integrated, which has been developed and held by the Polytechnic University of Madrid and AITEMIN (Association for Industrial Research and Development of Natural Resources). It also integrated a LAS control and an automated Märzhäuser platen, all monitored from a DELL workstation. High- and low-reflectance Ocean Optics patterns were used as a reference standard for the measurement of VNIR spectra. Aphelion software was used in image processing.

#### 3.2.2. X-ray Diffraction (XRD)

The X-ray diffraction (XRD) study was carried out to determine the mineralogical phases present in the investigated samples. A Rigaku Miniflex 600 X-ray diffractometer (Madrid, Spain) for qualitative and quantitative analyses was used. This equipment operates with an X-ray tube at 600 watts. It has a graphite monochromator and a standard scintillation counter. In addition, it has an automatic sampler with 6 positions. It has a HyPix-400 MF-2DHPAD detector (Madrid, Spain) and a ShapeFlex sample holder. It has SmartLab Studio II software (Madrid, Spain). It has an interface with a profile view, phase data view, 3D view and crystal structure view. The power requirement is 1ø, 100–240 v and 50/60 Hz. For this analysis, 500 milligrams per sample were weighed, ground up and screened up to 74 μ. One tablet for each sample was made in their respective sample holder moulds and then placed in the sample holder for analysis.

#### 3.2.3. X-ray Fluorescence (XRF)

An X-ray fluorescence (XRF) study was performed to determine the chemical compositions of the analysed samples. A Phillips fluorescence equipment model PW-1404 (Madrid, Spain) was used in the analysis. This equipment has a collimator to decrease the angle of divergence of the X-rays to control the offsets and to increase the reinforcement of the beam. The radiation intensity of the samples was 10–100 kV. A monochromator was used to isolate the measured radiation and to obtain an adequate wavelength. Six to eight grams of ground-up sample up to 74 μ were mixed with 1.5 mL of a solution composed of 250 cc of acetone and 12.5 gm of plastic to provide agglomerating properties to the sample and avoid possible collapses during pressing. This mixture was homogenised and allowed to dry at room temperature for 5 min. It was then placed inside an aluminium capsule 32 mm in diameter and pressed with a Herzog-branded press until a test pad of 5 cm in diameter was obtained. To determine the loss on ignition, 1 g of the original sample grounded up and sieved at 74 μ was taken. The sample was introduced into a Heraeus muffle at 1000 °C to remove the sulphates, carbonates, organic matter, water and other compounds. The percentage of loss on ignition (LOI) was applied to the results obtained in the spectrometer as a correction factor for the results.

#### 3.2.4. Scanning Electron Microscopy (SEM)

A Hitachi S-570 Scanning Electron Microscope (Madrid, Spain) was used in this research. The equipment was a Kevex 1728 analyser (Madrid, Spain), a BIORAD Polaron Division Carbon (Madrid, Spain) Evaporation Power Supply and a Polaron SEM Coating System. The resolution reached was 200 × 10^3^. In addition, the equipment had other components, such as: a semiconductor detector tube composed of silicon doped with lithium, a liquid nitrogen tank, a filament chamber, an electronic cannon, controls to insert and remove the sample from the high vacuum chamber and to vary the angles of the position of the sample, an interface module to visualise the image during the electronic scanning of the samples and two softwares, Winshell and Printerface, to manage the information of the analysed sample and to take microphotographs. For the performance of the test, the samples were previously reduced to 0.2–0.5 cm in diameter, then placed on a graphite adhesive tape and, then, in the sample holder. The samples were then covered with a layer of vacuum graphite. The samples were then placed in the sample holder of the high vacuum chamber of the electron microscope for analysis.

#### 3.2.5. Electrical Conductivity Test (ECT)

This method is based on the reaction experienced by a pozzolanic material within a saturated solution in Ca(OH)_2_. This method takes a measurement of the amount of Ca(OH)_2_ that reacts with the pozzolan sample in a normalised period of time. The principle of the method is based on the measurement of the electrical conductivity (EC) of the Ca(OH)_2_–pozzolan solution. During the trial, the variation of the concentration of Ca(OH)_2_ (CC) was monitored by measuring the EC, which is directly proportional to the Ca^2+^ ions present in the solution; it is necessary to know beforehand the correspondence between the conductivity (EC) and the concentration (CC). The ratio between the consumed Ca^2+^ (concentration of Ca^2+^ in time t) and the initial concentration is considered an index of pozzolanic reactivity; that is, the higher the consumption of Ca^2+^, the greater the pozzolanic activity of the sample. The conductance of the solution (SC) is the reciprocal of the electrical resistance (ER) (Ohm-L or Siemens in the international Systems of Measurements). Specific conductance (SC) is the conductivity, which is defined as the reciprocal of resistance in Ohm-L in 1 mL of liquid at a specific temperature. The unit of electrical conductivity (EC) is mS/cm. To perform the test, the samples were crushed, ground up and sieved to a particle diameter < 63 µm. A standard solution of Ca(OH)_2_, with a concentration of 10 g/L in distilled water, was prepared. It was stirred for 2 h and then left to stand for 24 h. Then, the solution was filtered and evaluated with HCl to find out the concentration (CC). The concentration was expressed in g/L and in mol/L. To build the calibration curve, 6 aliquots were prepared in distilled water at 10, 25, 40, 50, 75 and 90%, as well as a blank solution to determine the contribution of distilled water ions to the conductivity (EC). A conductivity measurement was made of both solutions, and their concentration was measured by comparison with the reference solution. A graph was made by relating the EC (*X*-axis) and the concentration (CC) (*Y*-axis), which permitted the calculation of the concentration of Ca^2+^. Once the calibration curve was obtained, the pozzolanic reaction was monitored by measuring the electrical conductivity from time 0 to 72 h. Then, 30 mL of a solution was prepared at 60% of the standard solution to which 3 g of sample were added and heated to 40 °C.

#### 3.2.6. Chemical Pozzolanicity Test (CPT)

The chemical pozzolanicity test (CPT) is based on the comparison of calcium ion, expressed as a hydroxide in calcium hydroxide, with the amount of ion that saturates a solution of equal alkalinity [57]. This solution also contained a mixture of portland cement (PC) and a pozzolan that partially replaced it. The behaviour of the solution was assessed after a normalised period of 8 and 15 days, respectively. The test procedure was as follows: 100 g of a sample of PC-AVT were taken using a sample divider. After this, it was sieved in a 150–125-µm sieve. The retained material was ground up until it passed through the aforementioned sieves. Then, 20 g of the mixture were taken and deposited in 100 mL of deionised water with an electrical conductivity ≤ 0.5 mS/cm at a temperature of 40 °C. The sample was stirred vigorously for 20 s and then left to stand for 8–15 days. The solution was then filtered for 30 s over a Buchner funnel, and double-dry filter paper was used. It was allowed to cool to room temperature. The total alkalinity of the solution was then determined, and the concentration of the hydroxyl [OH]^−^ and CaO ions was subsequently calculated using the following Equations (3) and (4):

Concentration of hydroxyl ions [OH]^−^:

(3) $${\left[\mathrm{OH}\right]}^{-}=\frac{1000\times 0.1\times V_{3}\times f_{2}}{50}=2\times V_{3}\times f_{2}$$

where V_3_ is the dissolution volume of HCl 0.1 mol/L used in titration, and f_2_ is the dissolution factor of HCl 0.1 mol/L.

CaO concentration:

(4) $$\left[\mathrm{CaO}\right]=\frac{1000\times 0.03\times V_{4}\times f_{1}}{50}=0.6\times V_{4}\times f_{1}$$

where V_4_ is the volume of EDTA (ethylenediaminetetraacetic acid) solution used in titration (mL), and f_1_ is the factor of the dissolution of EDTA.

#### 3.2.7. Mechanical Strength

This method was performed to determine the mechanical compressive strength of the prismatic mortar samples; the dimensions were 40 × 40 × 160 mm [60]. In the dosage of the mortar, a normalised sand (NS) Type CEN EN 196-1 was used; this sand is made of quartz of rounded grains, where the SiO_2_ content is 98%. The water absorption capacity was 3.5%. The granulometric distribution of normalised sand (NS) is listed in Table 2.

A portland cement (PC) Type 1, with resistance class 42.5 R was used in this research following the requirements indicated by the standard UNE-EN 196-1:2005 [60]. The chemical composition of cement was calculated in this research and is shown in Table 3.

In the preparation of the mixtures, two fundamental proportions were used: 75:25% and 70:30% PC-AVT, respectively. Two hundred and twenty-five grams of distilled water were used in both proportions: 75:25% and 70:30%. The procedure was developed as follows: one-part portland cement (PC), three-parts normalised sand (NS) and one-part deionised water (DW). The water/cement ratio (w/c) was 0.5 [60]. The specimens were placed inside a container with water at a temperature of 20 °C ± 1 °C. The temperature and relative humidity of the wet chamber were 20 °C ± 1 °C and 90%, respectively. The ages chosen to determine the mechanical compressive strength were 2, 7, 28 and 90 days [60].

## 4. Conclusions

The samples analysed had a complex mineralogical, petrological and chemical makeup, typical of the altered tuffs of volcanic origin. The tests carried out during this research indicated the presence of smectite (montmorillonite), mordenite, plagioclase, illite, gypsum, quartz, kaolinite and amorphous materials.

All the samples analysed were pozzolan, despite having a complex composition; thus, the objective of this research was achieved.

There was a solid agreement between the results obtained in all the tests carried out to establish the pozzolanic properties of the analysed samples; TFF-01 and TFF-03 were the most reactive with regards to TFF-02, which was due to a greater amount of mordenite, volcanic glass and clay minerals in the aforementioned samples.

The results of this research could be useful to recommend the industrial manufacturing of pozzolanic cements with the proportions 75:25% and 70:30%, since they provide practically the same mechanical strength; however, it should be noted that the 70:30% ratio contains less portland cement (PC), which is advantageous because of the lower degree of CO_2_ that would be emitted in the production process.

Finally, these results could be a useful guide for the integral use of altered volcanic tuffs (AVT) as highly valuable industrial rocks. In the specific case of the Los Frailes caldera, where the altered volcanic tuffs are the host rocks of the zeolitic mineralisation, it is recommended to use both materials together in the manufacturing of mortars, concretes and high-quality pozzolanic cements.

## Figures and Tables

**Table 1:** Chemical compositions of the samples investigated using XRF.

Sample	(% Weight)								LOI (%)
	SiO_2_	Al_2_O_3_	CaO	Na_2_O	K_2_O	MgO	Fe_2_O_3_	TiO_2_	
TFF-01	65.99	14.57	0.96	2.89	3.03	1.56	1.72	0.116	14.20
TFF-02	62.46	13.51	1.16	1.94	1.36	3.2	1.83	0.113	11.15
TFF-03	65.17	14.03	0.874	2.64	2.21	1.85	1.52	0.133	12.68

**Table 2:** Granulometric distribution of the normalised sand (NS) used in this research [60].

Square Mesh Dimensions (mm)	2.00	1.60	1.00	0.5	0.16	0.08
Residue Retained on Sieves (%)	0.00	7 ± 5	33 ± 5	67 ± 5	87 ± 5	99 ± 1

**Table 3:** Chemical composition of the portland cement (PC) used in this research.

Portland Cement (PC)	Compounds in Mass Percentage (% Weight)											LOI (%)	% Total
	S_i_O_2_	Al_2_O_3_	K_2_O	Na_2_O	MgO	Fe_2_O_3_	CaO	TiO_2_	SO_3_	MnO	P_2_O_5_		
	17.45	5.59	1.37	0.091	0.641	3.35	64.04	0.326	4	0.094	0.072	2.43	99.454

## Data Availability

Not applicable.

## Sample Availability

Samples of the compounds not applicable are available from the authors.

## References

[1] Mielenz R.C., Greene K.T., Schieltz N.C. (1951). Natural pozzolans for concrete. Econ. Geol..

[2] Altwair N.M., Kabir S. Green concrete structures by replacing cement with pozzolanic materials to reduce greenhouse gas emissions for sustainable environment. Proceedings of the American Society of Civil Engineers, 6th International Engineering and Construction Conference (IECC’6).

[3] Alhozaimy A., Fares G., Alawad O.A., Al-Negheimish A. (2015). Heat of hydration of concrete containing powdered scoria rock as a natural pozzolanic material. Constr. Build. Mater..

[4] Merida A., Kharchi F. (2015). Pozzolan concrete durability on sulphate attack. Procedia Eng..

[5] Fairbairn E.M.R., Americano B.B., Cordeiro G.C., Paula T.P., Toledo Filho R.D., Silvoso M.M. (2010). Cement replacement by sugar cane bagasse ash: CO_2_ emissions reduction and potential for carbon credits. J. Environ. Manag..

[6] Bakhtyar B., Kacemi T., Nawaz A. (2017). A Review on carbon emissions in Malaysian cement industry. Int. J. Energy Econ. Policy.

[7] Voit K., Zeman O., Janotka I., Adamcova R., Bergmeister K. (2020). High-durability concrete using eco-friendly slag-pozzolanic cements and recycled aggregate. Appl. Sci..

[8] Hua X., Shi C., Shi Z., Tong B., Wang D. (2017). Early age shrinkage and heat of hydration of cement-fly ash-slag ternary blends. Constr. Build. Mater..

[9] Sideris K., Justnes H., Soutsos M., Sui T., De Belie N., Soutsos M., Gruyaert E. (2018). Fly ash. Properties of Fresh and Hardened Concrete Containing Supplementary Cementitious Materials.

[10] Naik T.R., Asce F. (2008). Sustainability of concrete construction. Pract. Period. Struct. Des. Constr..

[11] Khan M.I., Alhozaimy A.M. (2010). Properties of natural pozzolan and its potential utilization in environmental friendly concrete. Can. J. Civ. Eng..

[12] Redondo-Vega J.M., Gómez-Villar A., Santos-González J., González-Gutiérrez R.B., Álvarez-Martínez J. (2017). Changes in land use due to mining in the north-western mountains of Spain during the previous 50 years. Catena.

[13] U.S. Geological Survey (2011). Mineral Commodity Summaries 2011. https://geoinfo.nmt.edu/staff/mclemore../teaching/imclass/documents/mcs2011.pdf.

[14] Walker R., Pavía S. (2011). Physical properties and reactivity of pozzolans and their influence on the properties of lime–pozzolan pastes. Mater. Struct..

[15] Cobîrzan N., Balog A.-A., Moşonyi E. (2015). Investigation of the natural pozzolans for usage in cement industry. Procedia Technol..

[16] Hussein K.M., Bouchra E.H., El Youbi M.S., Ahmed E. (2017). Development and study of physical, chemical and mechanical properties of a new formulation of cement of a varying percentage of natural pozzolan. J. Chem. Technol. Metall..

[17] Raggiotti B.B., Positieri M.J., Oshiro A. (2018). Natural zeolite, a pozzolan for structural concrete. Procedia Struct. Integr..

[18] Al-Zou’by J., Al-Zboon K.K. (2014). Effect of volcanic tuff on the characteristics of cement mortar. Cerâmica.

[19] Yu L., Zhou S., Deng W. (2015). Properties and pozzolanic reaction degree of tuff in cement–based composite. Adv. Concr. Constr..

[20] Biricik H. (2020). Pozzolanic activity of central Anatolian volcanic tuff and its usability as admixture in mortar. Adv. Cem. Res..

[21] Liguori B., Iucolano F., de Gennaro B., Marroccoli M., Caputo D. (2015). Zeolitized tuff in environmental friendly production of cementitious material: Chemical and mechanical characterization. Constr. Build. Mater..

[22] Balog A.A., Cobîrzan N., Aciu C., Iluţiu-Varvara A. (2014). Valorification of volcanic tuff in constructions and materials manufacturing industry. Proc. Technol..

[23] Najimi M., Sobhani J., Ahmadi B., Shekarchi M. (2012). An experimental study on durability properties of concrete containing zeolite as a highly reactive natural pozzolan. Constr. Build. Mater..

[24] Memon S.A., Arsalan R., Khan S., Yiu Lo T. (2012). Utilization of Pakistani bentonite as partial replacement of cement in concrete. Constr. Build. Mater..

[25] Kotwica L., Pichór W., Kapeluszna E., Różycka A. (2017). Utilization of waste expanded perlite as new effective supplementary cementitious material. J. Clean. Prod..

[26] Ulusu H., Yilmaz Aruntas H., Gencel O. (2016). Investigation on characteristics of blended cements containing pumice. Constr. Build. Mater..

[27] Baki V.A., Nay S., Erdoğdu Ş., Ustabaş İ. (2021). Pozzolanic properties of trachyte and rhyolite and their effects on alkali-silica reaction. Adv. Concr. Constr..

[28] Kwon Y.-H., Kang S.-H., Hong S.-G., Moon J. (2017). Intensified pozzolanic reaction on kaolinite clay-based mortar. Appl. Sci..

[29] Akacem M., Zentar R., Mekerta B., Sadok A., Omar H.M. (2020). Co-valorisation of local materials tuffs and dune sands in construction of roads. Geotech. Geol. Eng..

[30] Al-Zboon K., Al-Zou’by J., Abu-Hamatteh Z. (2019). Utilization of volcanic tuffs as construction materials. Jordanian J. Eng. Chem. Ind..

[31] Cherrak M., Bali A., Silhadi K. (2013). Concrete mix design containing calcareous tuffs as a partial sand substitution. Constr. Build. Mater..

[32] Ababneh A., Matalkah F. (2018). Potential use of Jordanian volcanic tuffs as supplementary cementitious materials. Case Stud. Constr. Mater..

[33] Lesovik R.V., Ageeva M.S., Shakarna M.I. (2013). Efficient binding using composite tuffs of the Middle East. World Appl. Sci. J..

[34] Baloga A.A., Cobîrzan N., Aciu C., Iluţiu-Varvara D.A. Valorification of volcanic tuff in constructions and materials manufacturing in industry. Proceedings of the 7th International Conference Interdisciplinarity in Engineering (INTER-ENG 2013).

[35] Rocholl A., Schaltegger U., Gilg H.A., Wijbrans J., Böhm M. (2018). The age of volcanic tuffs from the upper freshwater molasse (North Alpine Foreland Basin) and their possible use for tephrostratigraphic correlations across Europe for the Middle Miocene. Int. J. Earth Sci..

[36] Balegh B., Sellaf H., Hadjmostefa A. (2020). Effect of ceramic waste on mechanical and geotechnical properties of tuff treated by cement. Case Stud. Constr. Mater..

[37] Sarireh M. (2015). Optimum percentage of volcanic tuff in concrete production. Yanbu J. Eng. Sci..

[38] Capaccioni B., Cinelli G., Mostacci D., Tositti L. (2012). Long-term risk in a recently active volcanic system: Evaluation of doses and indoor radiological risk in the quaternary Vulsini Volcanic District (Central Italy). J. Volcanol. Geotherm. Res..

[39] Korkanç M., Solak B. (2016). Estimation of engineering properties of selected tuffs by using grain/matrix ratio. J. Afr. Earth Sci..

[40] Germinario L., Török Á (2020). Surface weathering of tuffs: Compositional and microstructural changes in the building stones of the medieval castles of Hungary. Minerals.

[41] Heap M.J., Farquharson J.I., Kushnir A.R.L., Lavallée Y., Baud P., Gilg H.A., Reuschlé T. (2018). The influence of water on the strength of neapolitan yellow tuff, the most widely used building stone in Naples (Italy). Bull. Volcanol..

[42] Woszuk A., Wróbel M., Franus W. (2020). Application of zeolite tuffs as mineral filler in warm mix asphalt. Materials.

[43] Sudo T. (1954). Clay mineralogical aspects of the alteration of volcanic glass in Japan. Clay Miner. Bull..

[44] Monecke T., Giorgetti G., Scholtysek O., Kleeberg R., Götze J., Hannington M.D., Petersen S. (2007). Textural and mineralogical changes associated with the incipient hydrothermal alteration of glassy dacite at the submarine PACMANUS hydrothermal system, eastern Manus Basin. J. Volcanol. Geotherm. Res..

[45] Costafreda J.L. (2008). Geología, Caracterización y Aplicaciones de las Rocas Zeolíticas del Complejo Volcánico de Cabo de Gata (Almería). Ph.D. Thesis.

[46] Heiken G., The Geological Society of America (2006). Tuffs. Their Properties, Uses, Hydrology and Resources.

[47] Soriano C., Riggs N., Giordano G., Porreca M., Conticellie S. (2012). Cyclic growth and mass wasting of submarine Los Frailes lava flow and dome complex in Cabo de Gata, SE Spain. J. Volcanol. Geotherm. Res..

[48] Oyarzun R., López García J.A., Crespo E., Lillo J. (2018). Neat stratigraphic and dynamic relationships between pyroclastic flow and ash-cloud surge deposits in the Cabo de Gata–Níjar Geopark, Almería, Spain. Int. J. Earth Sci..

[49] Christiansen M.U., Dymond B.Z. (2019). Effect of composition on performance of ground glass pozzolan. ACI Mater. J..

[50] Mark O.G., Ede A.N., Olofinnade O., Bamigboye G., Okeke S.C., Oyebisi O., Arum C. (2019). Influence of some selected supplementary cementitious materials on workability and compressive strength of concrete. A Review. IOP Conf. Ser. Mater. Sci. Eng..

[51] Arribas A. (1992). Las Mineralizaciones de Metales Preciosos de la Zona Central del Cabo de Gata (Almería) en el Contexto Metalogénico del Sureste de España. Ph.D. Thesis.

[52] Costafreda J.L., Martín D.A. (2021). Bentonites in Southern Spain. Characterization and applications. Crystals.

[53] Giorgetti G., Monecke T., Kleeberg R., Hannington M.D. (2006). Low-temperature hydrothermal alteration of silicic glass at the PACMANUS hydrothermal vent field, Manus basin: An XRD, SEM and AEM-TEM study. Clays Clay Miner..

[54] Rosell-Lam M., Villar-Cociña E., Frías M. (2011). Study on the pozzolanic properties of a natural Cuban zeolitic rock by conductometric method: Kinetic parameters. Constr. Build. Mater..

[55] Tironi A., Trezza M.A., Scian A.N., Irassar E.F. (2013). Assessment of pozzolanic activity of different calcined clays. Cem. Concr. Compos..

[56] Fernández R., Martirena F., Scrivener K.L. (2011). The origin of the pozzolanic activity of calcined clay minerals: A comparison between kaolinite, illite and montmorillonite. Cem. Concr. Res..

[57] (2006). Ensayo de puzolanicidad para cementos puzolánicos. Métodos de Ensayo de Cementos.

[58] Baykara H., Cornejo M.H., Murillo R., Gavilanes A., Paredes C., Elsen J. (2017). Preparation, characterization and reaction kinetics of green cement: Ecuadorian natural mordenite-based geopolymers. Mater. Struct..

[59] Perraki T., Kontori E., Tsivilis S., Kakali G. (2010). The effect of zeolite on the properties and hydration of blended cements. Cem. Concr. Compos..

[60] (2005). Determinación de resistencias mecánicas. Métodos de Ensayo de Cementos.

[61] Abd Elaty M.A.A. (2014). Compressive strength prediction of Portland cement concrete with age using a new model. HBRC J..

[62] Keppert M., Kobera L., Scheinherrová L., Doleželová M., Brus J., Černý R. (2020). Kinetics of pozzolanic reaction and carbonation in ceramic—Lime system: Thermogravimetry and solid-state NMR spectroscopy study. J. Build. Eng..

[63] Nwankwo C.O., Bamigboye G.O., Davies I.E.E., Michaels T.A. (2020). High volume Portland cement replacement: A review. Constr. Build. Mater..

[64] Zhang D., Zhao J., Wang D., Wang Y., Ma X. (2020). Influence of pozzolanic materials on the properties of natural hydraulic lime based mortars. Constr. Build. Mater..

[65] Senhadji Y., Escadeillas G., Khelafi H., Mouli M., Benosman A.S. (2012). Evaluation of natural pozzolan for use as supplementary cementitious material. Eur. J. Environ. Civ. Eng..

[66] Frias Rojas M., Sánchez de Rojas Gómez M.I. (2013). Artificial pozzolans in eco-efficient concrete. Eco-Efficient Concrete.

[67] Li L.G., Feng J.-J., Zhu J., Chu S.-H., Kwan A.K.H. (2021). Pervious concrete: Effects of porosity on permeability and strength. Mag. Concr. Res..

[68] Li L.G., Zheng J.Y., Ng P.-L., Kwan A.K.H. (2021). Synergistic cementing efficiencies of nano-silica and micro-silica in carbonation resistance and sorptivity of concrete. J. Build. Eng..

[69] Google Earth. https://earth.google.com/web/@36.77856752,-2.0702902,74.18166396a,7151.47016211d,1y,0h,0t.

